# Population Pharmacokinetics of Intravenous Artesunate: A Pooled Analysis of Individual Data From Patients With Severe Malaria

**DOI:** 10.1038/psp.2014.43

**Published:** 2014-11-05

**Authors:** S G Zaloumis, J Tarning, S Krishna, R N Price, N J White, T M E Davis, J M McCaw, P Olliaro, R J Maude, P Kremsner, A Dondorp, M Gomes, K Barnes, J A Simpson

**Affiliations:** 1 Centre for Epidemiology and Biostatistics, Melbourne School of Population and Global Health, University of Melbourne, Melbourne, Australia; 2 Mahidol-Oxford Tropical Medicine Research Unit, Faculty of Tropical Medicine, Mahidol University, Bangkok, Thailand; 3 Institute for Infection and Immunity, St. George's, University of London, London, UK; 4 Centre for Clinical Vaccinology and Tropical Medicine, Nuffield Department of Medicine, University of Oxford, Oxford, UK; 5 Centre for Tropical Medicine, Nuffield Department of Clinical Medicine, University of Oxford, Oxford, UK; 6 School of Medicine and Pharmacology, Fremantle Hospital, University of Western Australia, Fremantle, Australia; 7 World Health Organization, Genève, Switzerland; 8 Centre for Tropical Medicine, Nuffield Department of Medicine, University of Oxford, Oxford, UK; 9 Medical Research Unit, Albert Schweitzer Hospital, Lambaréné, Gabon; 10 Institute for Tropical Medicine, Department of Parasitology, University of Tübingen, Tübingen, Germany; 11 Faculty of Tropical Medicine, Mahidol University, Bangkok, Thailand; 12 Division of Clinical Pharmacology, Department of Medicine, University of Cape Town, Cape Town, South Africa

## Abstract

There are ~660,000 deaths from severe malaria each year. Intravenous artesunate (i.v. ARS) is the first-line treatment in adults and children. To optimize the dosing regimen of i.v. ARS, the largest pooled population pharmacokinetic study to date of the active metabolite dihydroartemisinin (DHA) was performed. The pooled dataset consisted of 71 adults and 195 children with severe malaria, with a mixture of sparse and rich sampling within the first 12 h after drug administration. A one-compartment model described the population pharmacokinetics of DHA adequately. Body weight had the greatest impact on DHA pharmacokinetics, resulting in lower DHA exposure for smaller children (6–10 kg) than adults. *Post hoc* estimates of DHA exposure were not significantly associated with parasitological outcomes. Comparable DHA exposure in smaller children and adults after i.v. ARS was achieved under a dose modification for intramuscular ARS proposed in a separate analysis of children.

Severe malaria kills ~660,000 people worldwide annually, the greatest burden of mortality occurring in young children.^1^ Early diagnosis and treatment with highly effective antimalarial drugs are critical in the management of acute malaria, since most deaths occur within the first 24 h of presentation.^2,3^ Two large multicenter randomized controlled trials have demonstrated conclusively the superior efficacy of parenteral artesunate (ARS) over intravenous (i.v.) quinine in reducing the mortality of severe falciparum malaria. ARS resulted in a 35% relative reduction in mortality in patients of all ages in the South East Asian Quinine Artesunate Malaria Trial study (10 centers in four Asian countries)^4^ and 23% in the African Quinine Artesunate Malaria Trial study of African children (11 centers in nine African countries).^5^

Based on these studies, i.v. ARS is now recommended as the first-line treatment for severe malaria for adults and children worldwide.^6^ Most guidelines recommend a single dose followed by a second dose at 12 h and then every 24 h until oral treatment can be tolerated. Although this dosing strategy appears efficacious, it is based on clinical trials rather than pharmacokinetic (PK)–pharmacodynamic (PD) assessments. A recent population PK analysis of ARS and dihydroartemisinin (DHA; the active metabolite of ARS) concentrations, after intramuscular (i.m.) administration of ARS (i.m. ARS) in African children with severe malaria, suggests that DHA exposure is reduced in smaller children (weighing 6–10 kg).^7^ A dosing regimen based on weight bands was found to achieve comparable DHA exposure levels in smaller and larger children, and indicates that the i.m. ARS for children weighing 6–10 kg should be between 2.7 and 3.3 mg/kg.^7^

The life-saving benefit of ARS in severe malaria results from its broad stage-specific activity over the 48 h life cycle of the parasite in the red blood cell. ARS kills the circulating ring-stage parasites before they can mature,^8,9^ thereby preventing deaths resulting from microvascular obstruction in the vital organs.^10,11,12^ Recent reports from South-East Asia indicate delayed parasite clearance for patients administered oral ARS, signaling the emergence of artemisinin-resistant *Plasmodium falciparum*.^13,14,15^ Mathematical modeling of data from this region^16^ suggests that the killing rate of ARS on the ring-stage parasites is reduced. This reduction in ring-stage killing results in a greater proportion of parasites maturing in the erythrocytes, which in turn would lead to more sequestration in the capillaries, end organ damage, and ultimately death. A possible strategy to overcome this reduction in the killing rate of ARS is to administer higher doses or give ARS more frequently. However, evidence from a clinical trial conducted in western Cambodia found that patients given 6 mg/kg of oral ARS daily for 7 days had an increased risk of neutropenia compared with those given 2 or 4 mg/kg.^17^

Based on evidence of emerging resistance and dose-dependent toxicity of ARS, it is crucial that the recommended dose and frequency of i.v. ARS dose is optimized from an understanding of the PK-PD relationships. In this study, we have compiled the largest dataset (as of May 2014) of ARS and DHA concentrations and parasite counts from adults and children with severe malaria and treated with i.v. ARS, developed a population PK model, identified clinical covariates that explain the variability in observed DHA PK profiles, and examined how DHA exposure varies with dosing regimens. The association between parasitological outcomes and *post hoc* estimates of DHA exposure for each patient was also examined.

## Results

### Clinical details

Data were collected from six studies (five published^18,19,20,21,22^ and one unpublished by WHO, but a subset of this study consisting of 12 Thai patients administered intrarectal ARS (i.r. ARS) was published in Simpson *et al.*^23^) conducted in Africa and Asia, of adults or children with severe or moderately severe malaria (**Table 1**). Four subgroup analyses were performed because the same set of covariates was not measured in each of the six studies. Subgroup 1 involves covariates measured in all six studies, subgroup 2 includes studies with measures of respiratory distress, and subgroups 3 and 4 include covariates measured only in children and adults, respectively. **Table 2** summarizes the distribution of the covariates.

In subgroup 1, 266 patients aged from 6 months to 65 years were included in the analysis, of whom 48% (128/266) had a documented fever at presentation. Children were more likely to present with anemia (hemoglobin < 5 g/dl; 20/195, 8.3%) than adults (2/71, 2.8%; *P* = 0.12) and have a higher baseline parasitemia (median (range) 155,099 (397–1,870,264) vs. 114,520 (80–1,145,000)/µl whole blood, respectively; *P* = 0.001). A total of nine patients died, and these deaths occurred in three of the six studies.^19,21,22^

### Population pharmacokinetic analysis

There was limited ARS concentration data available (**Supplementary Figure S1**), precluding formal PK modeling. Nonlinear mixed-effects (NLME) modeling of the DHA concentration data from each study separately (i.e., referred to as a meta-analytic approach—see “Methods” for details) identified the one-compartment PK model assuming i.v. bolus dose administration and additive error on the natural log scale as providing an adequate fit to the data. Forest plots of study heterogeneity additionally found that the estimates of population mean clearance (CL) and volume of distribution (V) tended to be lower in the second compared with the first period of the crossover trials (**Supplementary Figure S2**), which indicates that patients who received i.v. ARS in the second period tended to have higher drug exposure. The visual predictive checks (VPCs) of the two-level models and posterior predictive check (analogous to a VPC, see **Supplementary Information 3**) of the three-level Bayesian model used to investigate whether modeling between-study variability improves the predictive properties of the model are presented in **Supplementary Figure S3**. The posterior predictive check of the three-level model illustrates that this model tends to overpredict the between-patient variability in the pooled dataset, whereas the VPCs of the two-level models indicate that these models capture the between-patient variability more accurately. Based on these checks, it was decided to proceed with covariate selection without explicitly modeling between-study differences.

The covariates in each of the four subgroups (**Table 2**) were examined for association with population mean PK parameters using stepwise covariate selection (see “Methods” for details). Note all models included an allometric function of body weight on both the population mean CL and V and an additional age maturation function on the population mean CL (see **Supplementary Information 4**), unless otherwise stated. Since none of the covariates in subgroups 2–4 were found to be associated with the PK parameters (see **Supplementary Table S1**), the following analysis is restricted to subgroup 1. The parameter estimates for the final model fitted to the subgroup 1 data are presented in **Table 3**. In subgroup 1, the population mean V for men was estimated to be 14% lower (95% confidence interval (CI): 24–3% lower) compared with women (population mean V for men, 9.9 l and for women, 11.6 l), implying that men tend to achieve a higher maximum drug concentration (*C*_max_). However, since the population mean CL was the same for men and women, the total drug exposure (area under the curve (AUC)) for both sexes should be the same. The between-patient variability reduced substantially after inclusion of body weight as an allometric function on both population mean CL and V (from 91–60% for CL and 86–45% for V; change in objective function value (ΔOFV) of 245) and an additional age-related enzyme-maturation effect on CL (decreased to 59%; ΔOFV of 9.3), but inclusion of sex did not further reduce the between-patient variability (however, ΔOFV of 6.9).

**Figure 1:**
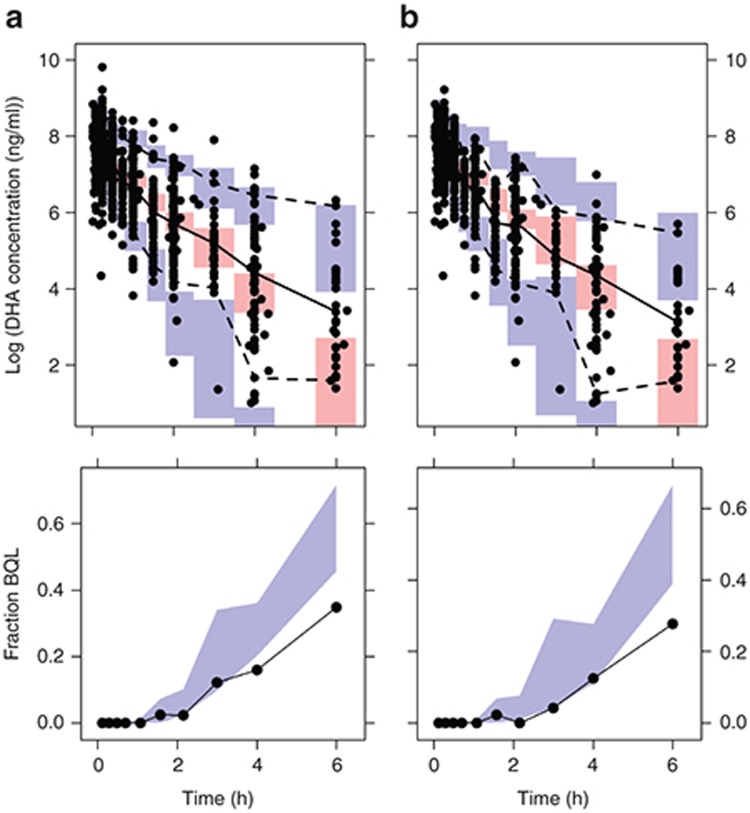
Visual predictive checks (VPCs) of final models fitted to (**a**) the PK dataset including patients in period two of the crossover trials (total *n* = 266) and (**b**) excluding patients in period two of the crossover trials (total *n* = 223). In the upper panel, the solid black circles are the observed log_e_ DHA plasma concentration (ng/ml) above the respective limit of quantification for each study (see **Supplementary Table S4**), and the following are plotted for bins across the independent variable time: 50th (solid black line), 2.5th, and 97.5th (dashed black lines) percentiles of the observed concentrations; and simulation-based 95% confidence intervals for the predicted 50th (pink region), 2.5th, and 97.5th (blue regions) percentiles. In the lower panel, the solid black line is the observed fraction of DHA concentrations (at each time point of sample collection) below quantification limit (BQL), and the blue region is the simulation-based 95% confidence interval for the predicted fraction of BQL DHA samples. DHA, dihydroartemisinin; PK, pharmacokinetic.

The sensitivity of stepwise covariate model selection to the exclusion of patients in period two of the crossover trials was also examined (**Table 3**). The exclusion of these patients resulted in stepwise covariate model selection identifying hemoglobin and body temperature to be associated with the population mean CL, in addition to the association between sex and population mean V. In patients receiving i.v. ARS at baseline, CL was decreased by 4% per unit (g/dl; 95% CI: 6% decrease to 1% decrease) increase of hemoglobin and increased by 7.0% per unit (°C; 95% CI: 2% increase to 12% increase) increase in body temperature. VPCs of the final model fitted to the dataset including patients in period two of the crossover trials, and excluding these patients indicate these models exhibit no model misspecification (**Figure 1**).

The simulation of DHA exposure after an i.m. ARS dose of 2.4 mg/kg described in Hendriksen *et al.*^7^ found that DHA exposure for smaller children (6–10 kg) was lower than that in larger children (21–25 kg); an adjusted dose regimen was found to rectify this difference. To examine whether DHA exposure after i.v. ARS, administered as the standard 2.4 mg/kg dose or according to the proposed weight-band dose regimen in Hendriksen *et al.*^7^ , exhibits a similar relationship with body weight, we replicated the Hendriksen *et al.*^7^ simulation using our final population PK model. DHA exposures (area under the curve from 0 to 12 h (AUC_0–12h_) h × ng/ml) after the standard 2.4 mg/kg dose were simulated at each body weight level (1,000 simulations each at 1 kg intervals from 6 to 25 kg; see **Supplementary Information 1** for details). Children with body weight between 6 and 10 kg showed a reduction in geometric mean DHA exposure of 13.7% (95% CI: 11.7–15.6%; *P* < 0.001) compared with children with body weight between 21 and 25 kg. The median (25th to 75th percentile) DHA exposure following a dose of 2.4 mg/kg was: 2,077 (1,403–3,124) h × ng/ml in the 6–10 kg patients; 2,127 (1,442–3,063) h × ng/ml in the 11–15 kg patients; 2,122 (1,443–3,094) h × ng/ml in the 16–20 kg patients; and 2,426 (1,669–3,517) h × ng/ml in patients 21–25 kg.

**Figure 2:**
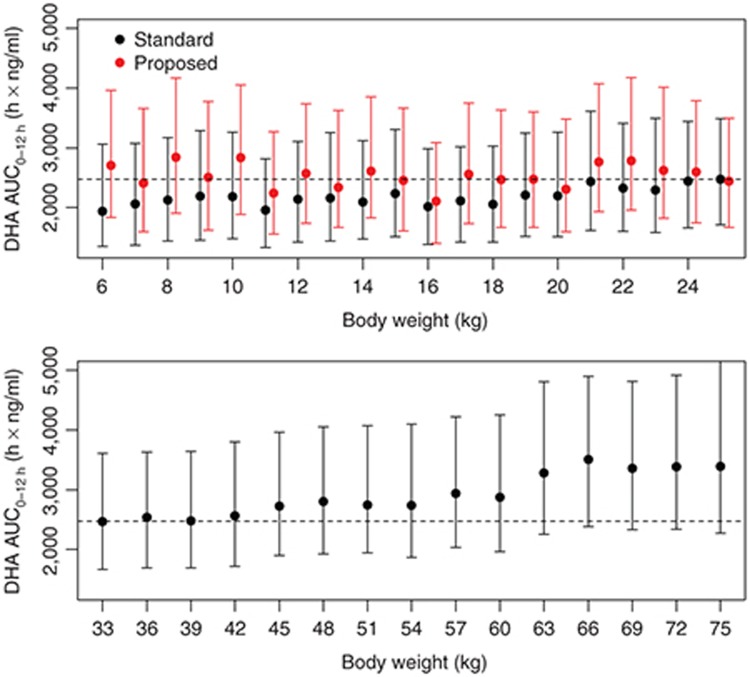
Simulated total first dose exposures (AUC_0–12h_) of DHA after the standard 2.4 mg/kg dosing in children at different body weights (black) and the adjusted dosing regimen proposed in Hendriksen *et al.*^7^ (red). Solid circles represent the median, and the error bars indicate the 25th and 75th percentiles of the 1,000 simulations at each body weight. The dashed line is the median exposure for the 25 kg weight group after the standard 2.4 mg/kg dose. DHA, dihydroartemisinin.

The results were consistent with those presented in Hendriksen *et al*.^7^ and suggest that smaller children need higher weight-adjusted doses of i.v. ARS to attain DHA exposures similar to children with higher body weights. The adjusted dosing regimen proposed in Hendriksen *et al.*^7^ for i.m. ARS appears to be applicable to i.v. ARS, as it results in similar exposures in all weight bands after the first dose of i.v. ARS (**Figure 2**).

A similar simulation was performed for patients older than 16 years and weighing between 33 and 75 kg (see **Supplementary Information 1** for details). The lower panel of **Figure 2** indicates that DHA exposure after the standard 2.4 mg/kg dose in patients weighing between 33 and 76 kg is comparable to DHA exposures achieved by children receiving the adjusted dosing regimen proposed in Hendriksen *et al.*^7^ (**Figure 2**, upper panel, red).

### Association between *post hoc* individual PK estimates and PD measures

The PD analysis was restricted to the 142 patients receiving a dose of 2.4 mg/kg i.v. ARS at baseline. The median parasite clearance half-life (time needed for parasitemia to reduce by half)^24^ was 3.1 h (interquartile range: 2.4–3.8; range: 0.9–6) with 25% (14/55) of patients clearing 90% of their baseline parasitemia by 12 h (PC90 at 12 h). Although none of the *post hoc* PK estimates were associated with the parasite clearance half-life, an association between *post hoc* DHA V and PC90 at 12 h was found (**Table 4**)—a doubling of V was associated with a 0.28-fold decrease in the odds of PC90 at 12 h (95% CI: 0.07–1.11; *P* = 0.07). Since many patients did not have parasite measurements recorded at 12 h, we performed a sensitivity analysis where we imputed parasite count at 12 h for patients with at least three parasite counts recorded in the first 24 h, including one of these measurements post 12 h (see “Methods,” subsection “Pharmacodynamic measures” for details of imputation). Imputation allowed PC90 at 12 h to be calculated for an additional 53 (for a total of 108) of the 142 patients. Our findings in **Table 4** did not change materially on inclusion of the additional imputed PC90 at 12 h (e.g., odds ratios after inclusion of imputed PC90 at 12 h were: 1.07 for clearance; 0.38 for volume; 0.77 for AUC_0–12h_; and 2.14 for *C*_max_).

## Discussion

In the present study, a population PK analysis of the largest pooled dataset of DHA drug concentrations to date from severe malaria patients has been performed. A one-compartment PK model provided the best fit to the pooled DHA concentration–time data from three studies of nonpregnant South-East Asian adults and three studies of African children. Body weight was found to be the key covariate influencing the PK of DHA. No correlation between the *post hoc* individual estimates reflecting DHA drug exposure and the PD outcomes (parasite clearance half-life and PC90 at 12 h) was observed.

The ARS PK data were not modeled because many of the ARS concentrations were below the level of detection as early as 2 and 4 h post i.v. ARS administration. This is a function of study design as the half-life of ARS is much shorter than this, while prevention of *ex vivo* metabolism of ARS to DHA after sample collection by using an enzyme (esterase) inhibitor such as fluoride was not done in most studies. A 100% conversion of ARS into the metabolite, DHA, was assumed (i.e., DHA is the sole metabolite of ARS).^18^ This was thought reasonable because the maximum concentration of DHA occurred shortly (i.e., median 0.5 min) after i.v. ARS administration for a study with rich and early sampling within the first hour.^22^ A limitation of not being able to model the ARS PK data, and additionally, assuming 100% conversion of ARS to DHA, is overprediction of maximum DHA concentrations. However, the VPC demonstrated that the model captured the DHA concentration data adequately. Of note, a moderate correlation between the *post hoc* individual CL and V estimates was observed. Such a correlation is not typically observed for drugs administered i.v. and may be an artifact of not modeling the conversion of ARS into DHA (e.g., using a parent-metabolite model).

The individual DHA PK datasets comprising the pooled dataset ranged in sample size from 6 to 157 patients and were of homogenous sample populations. Collectively, there were 266 or 223 patients, depending on whether patients in the second period of the crossover trials were included or excluded from the analysis, providing reasonable statistical power to identify key patient characteristics. There were a number of study-specific characteristics that could potentially be sources of between-study variability: criteria used to diagnose severe malaria varied, patients with severe and moderately severe malaria were included; study design, crossover and parallel designs; sample preparation and measurement of variables (e.g., drug concentration, parasite counts, and clinical variables)^25,26^ were likely to be inconsistent across studies due to differences between sample preparation, preassay extraction, drug assays, and technicians. Our examination of between-study heterogeneity revealed that DHA exposure tended to be higher in the second period of the crossover trials (i.e., after patients have received two antimalarial treatments) compared with period one (following an initial treatment). Reduced parasitemia at the time of i.v. ARS administration in period two of the crossover trials (~12 h) does not appear to explain this finding; baseline parasitemia was not found to be statistically significantly associated with either of the population mean PK parameters in either the analysis including or excluding patients in period two of the crossover trials. Evidence of this increase in DHA exposure during convalescence was also seen in previous published PK analyses of the individual studies included in our pooled analyses.^20,22,23^ Conversely, DHA exposure following oral ARS in patients with uncomplicated malaria has been observed to decrease during convalescence.^27,28^ In the acute phase of severe malaria, parasite-mediated drug decomposition may occur. Another possible contributor is that greater hemolysis in the acute phase of severe malaria may result in greater *ex vivo* decomposition (differentially affecting lower drug concentrations) and artifactually accelerating the apparent decline in plasma concentrations^26^ unless samples are collected into tubes containing enzyme (esterase) inhibitors. Interestingly, our pooled analysis revealed that despite improvements in the assay methodology to measure plasma drug concentrations, improving from earlier (1996–2001, mostly high-performance liquid chromatography) to later studies (2006–2008, liquid chromatography–mass spectrometry), little change in the magnitude of the within-patient variability was observed (residual error: ~35%).

Median (range) *post hoc* estimates of DHA *C*_max_, AUC_0–12h_ h × ng/ml, and elimination half-life (*t*_1/2_) derived from our pooled PK model (2,946 ng/ml (1,148–6,501); 2,560 h × ng/ml (799–8,525); and 0.63 h (0.27–1.45), respectively) are consistent with those from the six individual pooled studies (medians range from 1,592—3,011 ng/ml for *C*_max_; 923–2,541.2 h × ng/ml for AUC; 0.33–0.67 h for *t*_1/2_) and an additional study of i.v. ARS in 14 Ugandan adults (median *C*_max_ 3,140; AUC: 3,492 h × ng/ml; *t*_1/2_: 1.31 h).^29^ Additionally, DHA PK summary measures from studies of ARS administered i.v. to healthy adult volunteers (medians range from 1,286—1,507 ng/ml for *C*_max_; 1,850 h × ng/ml for AUC; 0.88–1.15 h for *t*_1/2_) and adults with uncomplicated malaria (medians range from 2,648—2,758 ng/ml for *C*_max_; 2,377–2,872 h × ng/ml for AUC; 0.67–0.98 h for *t*_1/2_) reviewed in Morris *et al.*^30^ are also contained in the range of *post hoc* estimates derived from our pooled PK model. There is evidence that lower maximum concentrations for DHA are achieved after i.m. administration compared with i.v. administration, but estimates of DHA exposure are generally similar due to the high bioavailability and rapid absorption of i.m. ARS.^7,30^ In a recent study of i.m. ARS in African children with severe malaria, the median (range) estimates of DHA *C*_max_, AUC, and *t*_1/2_were 547 ng/ml (284–890), 890 h × ng/ml (297–2,510), and 0.427 h (0.145–1.18), respectively, lower than those derived from our pooled PK model for DHA after i.v. ARS.^7^

All covariates were deemed important *a priori*, and only age, body weight, sex, body temperature, baseline parasitemia, and hemoglobin were available for all six studies. Subgroup analyses were performed to investigate the other covariate effects, such as many of the laboratory variables (e.g., bilirubin) that were only measured in adults, thereby limiting our statistical power to detect PK–covariate relationships. The most significant covariate identified in our pooled PK analysis of adults and children was body weight. This finding has also been reported in other population PK studies of oral, i.r., and i.m. ARS in pediatric^7,28,31^ and mixed adult–pediatric populations.^23^ DHA exposure predicted from our pooled PK model after the standard 2.4 mg/kg dose of i.v. ARS was found to be lower for smaller children (6–10 kg) than that for larger children and adults (≥25 kg), similar to findings in Hendriksen *et al.*^7^ for DHA exposure predicted after the standard 2.4 mg/kg dose of i.m. ARS. The weight-band dosing regimen recommended in Hendriksen *et al.*^7^ for i.m. ARS is also predicted to improve DHA exposure for small children after i.v. ARS.

Exclusion of patients in period two of the crossover trials influenced covariate selection and resulted in associations between population mean CL and the covariates hemoglobin and body temperature. After controlling for body weight, the population mean V for men was lower compared to women, and lower hemoglobin concentrations and higher body temperatures were associated with reduced DHA exposure levels. Sex has also been reported to be associated with increased mean CL/F by 1.14-fold for a male compared with a female in a population PK study of i.r. ARS in a mixed adult–pediatric population.^23^ An explanation for the lack of observed association between hemoglobin and temperature with CL when patients who received i.v. ARS in the second period of the crossover trials are included is that hemoglobin and temperature values will have improved from baseline by the time these patients receive i.v. ARS in period two of the trial. Although the evidence of association was weak, the direction of effect between hemoglobin and temperature with CL was still the same as that observed in the analysis with period two patients excluded, just of a lower magnitude.

We did not observe any relationship between DHA exposure and the parasitological outcomes, parasite clearance half-life (determined using the PCEstimator)^24^ and PC90 at 12 h. This may be partly due to lack of statistical power. These analyses excluded patients who received i.v. ARS in the second period of the crossover trial (*n* = 43) and were not given an i.v. ARS dose between 2 and 3 mg/kg (*n* = 81), and patients whose parasitemia vs. time profiles did not allow determination of parasite clearance half-life (*n* = 27) or did not have parasitemia recorded 12 h posttreatment (*n* = 87). A lack of correlation between DHA exposure and the parasitological outcomes has also been observed for i.m. ARS in a pediatric population (also low statistical power, *n* = 70)^7^ and for i.r. ARS in mixed adult–pediatric populations (a larger study but very noisy data and sparse sampling).^23^ The original studies included in the pooled analysis were not designed (or were underpowered) to detect clinically important changes in parasitological outcomes. The power was further reduced by the inability to determine parasitological outcomes in all 142 patients who received 2.4 mg/kg of i.v. ARS at baseline. However, the estimates in **Table 4** are consistent with a doubling of DHA AUC_0–12h_ and *C*_max_ decreasing the parasite clearance half-life by 0.17 h (~10 min) and 0.12 h (~7 min), respectively.

In conclusion, DHA exposure was lower for smaller children (6–10 kg) compared with adults. The findings from this pooled analysis of severe malaria patients receiving i.v. ARS support the dose adaption previously recommended in a separate analysis of children receiving i.m. ARS. The finding in Hendriksen *et al.*^7^ and in the current study that DHA exposure is lower in smaller children compared to larger children and adults suggests that the current dose may be suboptimal for smaller children. We were unable to quantify the clinical importance of this finding, but recommend dose finding studies, designed to detect clinically important changes in parasitological outcomes, should be performed, and the dose adaption proposed in Hendriksen *et al*.^7^ (and supported by this study) would be a suitable candidate for comparison to the current dosing regimen.

## Methods

### Study population, study design, dosing, and sampling

Details of the study population, design, and dosing for each of the six studies included in the pooled analysis are provided in **Table 1**. The criteria chosen by each study to define severe malaria is provided in **Supplementary Table S2**. Patients were considered as having moderately severe malaria if they did not have any of the features of severe malaria (see **Supplementary Table S2**) and could not be given oral therapy because of nausea, vomiting, or confusion. The blood sampling for determination of ARS and DHA concentrations and parasite counts is summarized in **Table 5**.

### Drug measurements

ARS and DHA plasma concentrations from each study were measured by different laboratories using either high-performance liquid chromatography with electrochemical detection or mass-spectrometry detection (**Supplementary Table S3**). The limit of quantification and limit of detection varied across the studies where the earlier studies had limit of quantifications of 50 ng/ml (limit of detections of 5–20 ng/ml) compared with around 2 ng/ml (0.3–0.6 ng/ml) in the more recent studies (**Supplementary Table S3**).

### Statistical analysis

*Pharmacokinetic modeling.* The analysis focused exclusively on the dynamics of DHA concentrations assuming complete *in vivo* conversion of ARS to DHA.^18^ This was justified since the maximum DHA concentrations were observed within minutes following ARS dosing. The administered dose of DHA (µg) was calculated using the relative difference in molecular weights for DHA and ARS: 284/384 (ARS to DHA conversion factor) × ARS dose (µg). Venous plasma concentrations were analyzed as natural logarithms using NLME modeling in NONMEM version 7.2 (ICON Development Solutions, Ellicott City, MD). The proportion of data below the limit of quantification is given in **Supplementary Table S4** and were modeled as censored data using the M3 method.^32^ Model selection was based on the OFV and either VPCs^33,34^ or posterior predictive checks^35^ generated from 1,000 simulated replicates of the pooled PK data. The bootstrap as implemented in Perl-speaks-NONMEM^36,37^ was used to calculate SEs and 95% CIs for the estimates of the population PK parameters and interindividual variability. The NONMEM code used to produce the results in **Table 3** is provided in the section “NONMEM code: IV-ARS pooled pharmacokinetic analysis” of the **Supplementary Information**.

*Between-study variability.* A meta-analytic approach was used to build the NLME model for the pooled DHA PK profiles. The purpose of these separate NLME model analyses was to determine whether the same structural and error models are suitable for each study and period of the crossover trials, and to visually compare the population mean PK parameter estimates derived for each study using a forest plot (see **Supplementary Information 2**). Between-study differences were also examined using NLME models (see **Supplementary Information 3** and **Supplementary Table S5**).

*Stepwise covariate selection.* Four subgroup analyses were performed because the same set of covariates was not measured in all studies; the covariates and study populations investigated in each subgroup are summarized in **Table 2**, and the stepwise covariate model procedure implemented in Perl-speaks-NONMEM was used to determine whether the covariates listed in **Table 2** were statistically significantly associated with the population PK parameters (see **Supplementary Information 4** for more details).

*Pharmacodynamic measures.* The pharmacodynamic measures selected *a priori* were: parasite clearance half-life (obtained from WorldWide Antimalarial Resistance Network's online Parasite Clearance Estimator)^24^ and the binary measure PC90 at 12 h (i.e., 90% of baseline parasitemia cleared at 12 h—yes (1), no (0)). These measures were derived for each individual from peripheral blood smears collected every 4–6 h (see **Supplementary Table S6** for parasitemia measurement details) and box plots of the baseline parasitemia for each study are presented in **Supplementary Figure S4**. Associations between the pharmacodynamic measures and *post hoc* individual estimates of DHA CL, V, AUC, and *C*_max_ were investigated using multivariable linear regression for parasite clearance half-life and logistic regression for PC90 at 12 h. The *post hoc* individual estimates of CL, V, AUC, and *C*_max_ were log_2_ transformed and included separately in each regression model. All regression analyses were adjusted for age (years), sex (male (1); female (0)), weight (kg), and baseline parasitemia (log_e_ transformed). These statistical analyses were performed using STATA Version 12 (StataCorp, College Station, TX). Single mean imputation of the parasite counts at 12 h was performed as follows: step 1, separate linear regressions including time as a linear and quadratic variable (i.e., time^2^) were fitted to each patient's parasite count (log_e_ units) vs. time profile; step 2, parasite count at 12 h was imputed using the estimated parameters derived in step 1; and step 3, steps 1 and 2 (i.e., imputation of 12-h parasite count) were only performed for patients with at least three parasite counts recorded in the first 24 h, including one of these measurements post-12 h.

## Author Contributions.

S.G.Z. and J.A.S. wrote the manuscript. J.A.S., R.N.P., S.K., N.J.W., and K.B. designed the research. S.K., P.K., A.D., R.J.M., M.G., P.O., T.M.E.D., and K.B. performed the research. S.G.Z., J.T., J.M.Mc.C., and J.A.S. analyzed the data.

## Conflicts of Interest.

S.K. is an (unpaid) advisor to Cipla and a consultant to Merck Serono for discovery of new antimalarials. The other authors declared no conflict of interest.

## Study Highlights


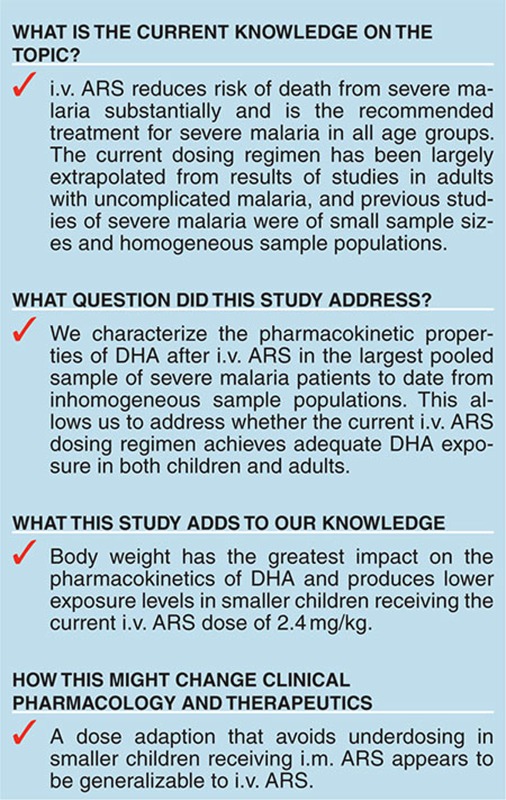


## Figures and Tables

**Table 1:**
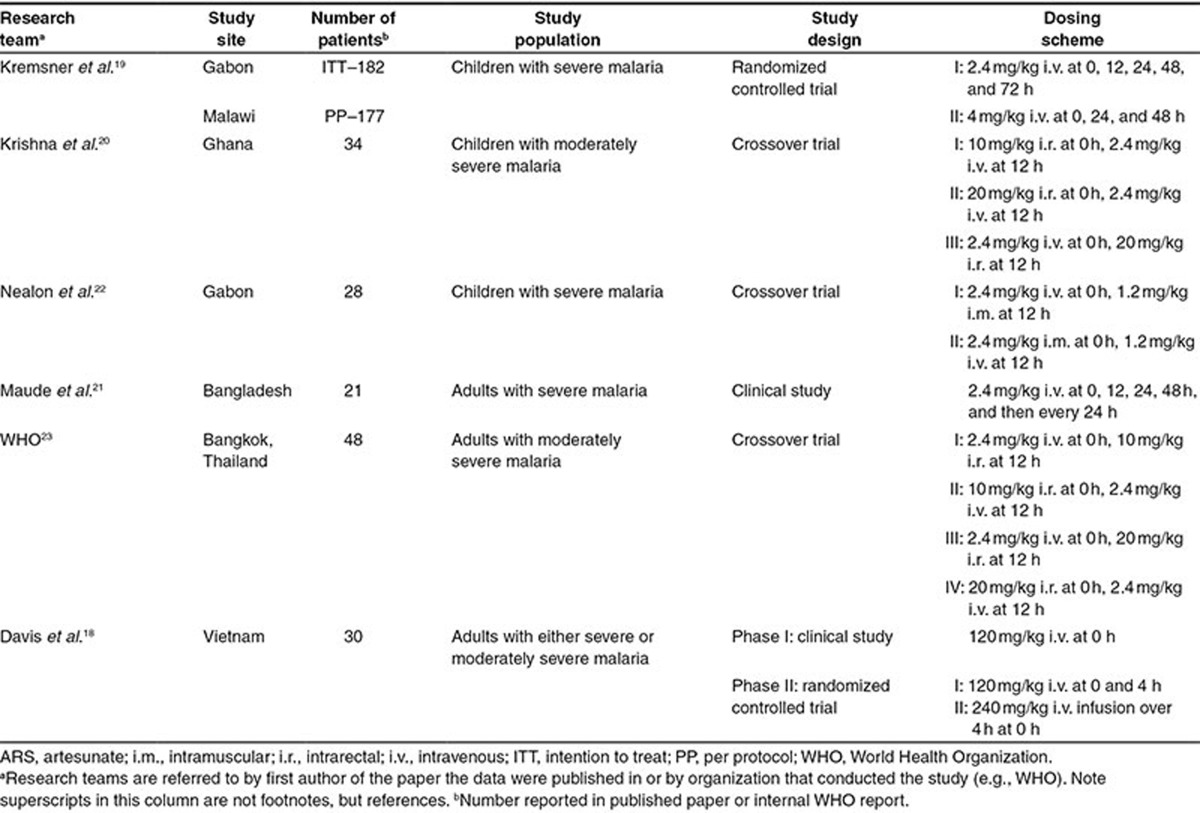
Study site, number of patients, study population, study design, and ARS dosing scheme(s) for all routes of ARS administration (i.v., i.r., and i.m.) and each study contributing data to the pooled analysis

**Table 2:**
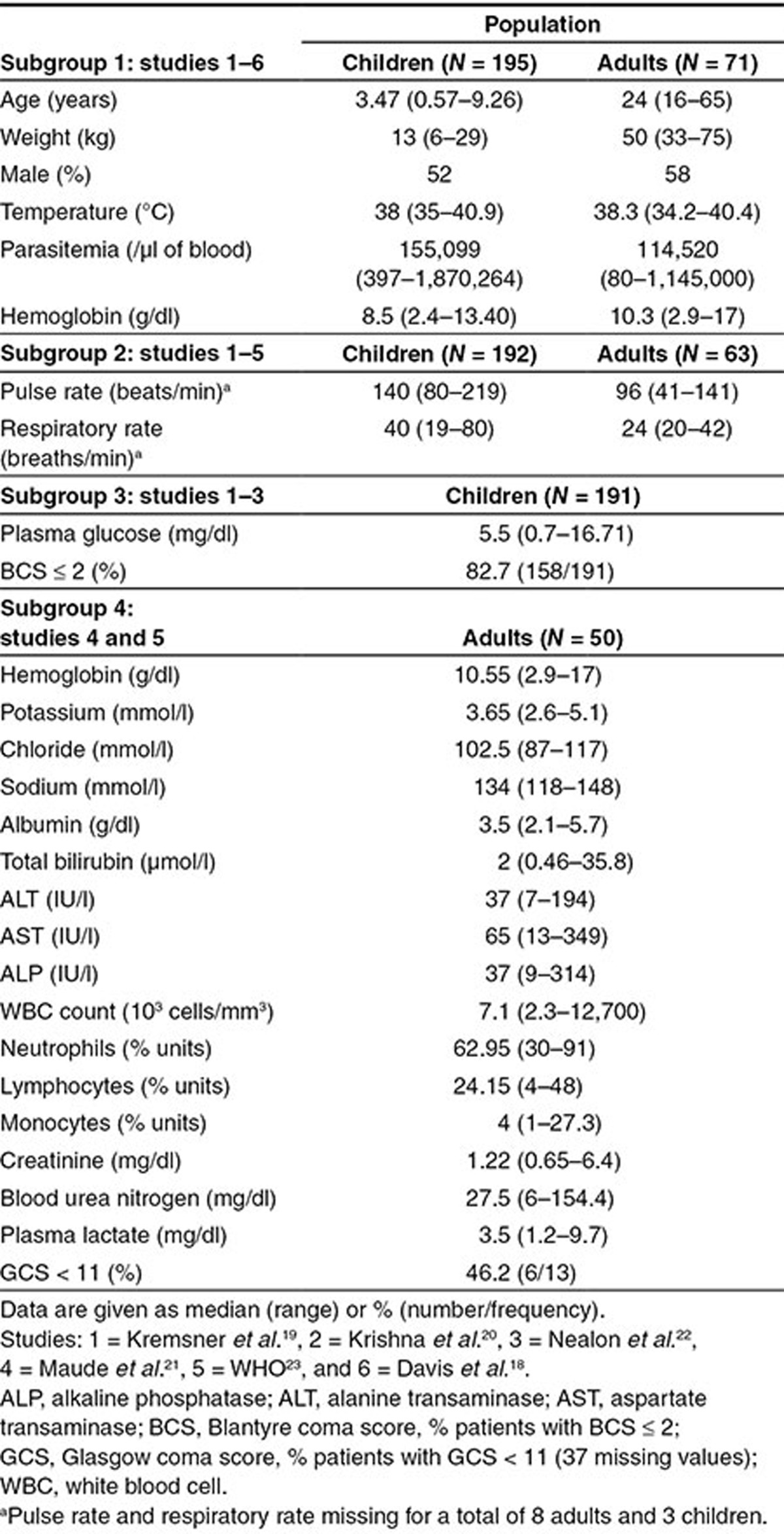
Study populations and distribution of baseline demographic, clinical, and laboratory characteristics of children and adults examined in each of the four subgroups

**Table 3:**
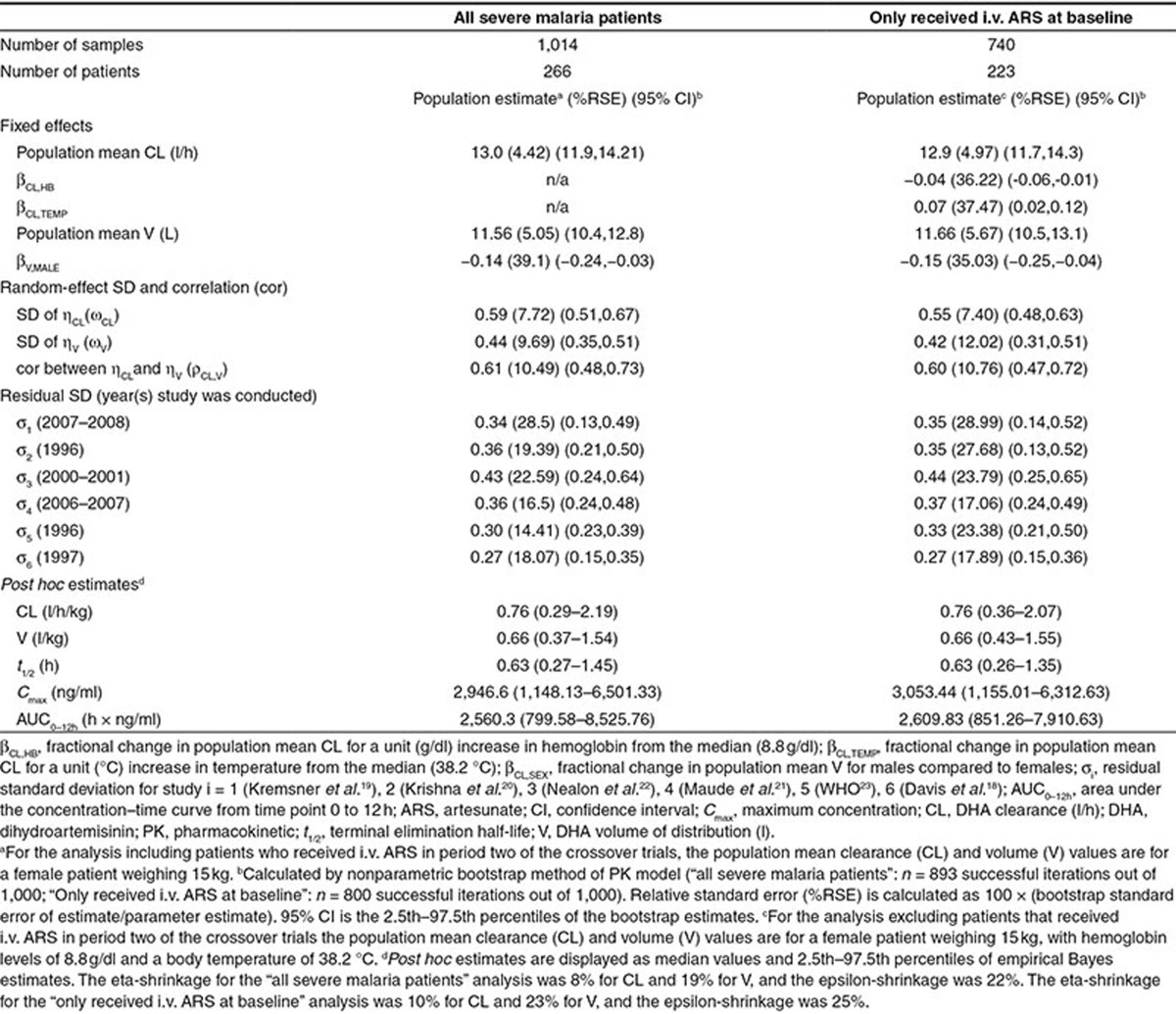
Parameter estimates of the final model describing the population pharmacokinetics of dihydroartemisinin in all severe malaria patients and for the subpopulation of patients who only received i.v. ARS at baseline

**Table 4:**
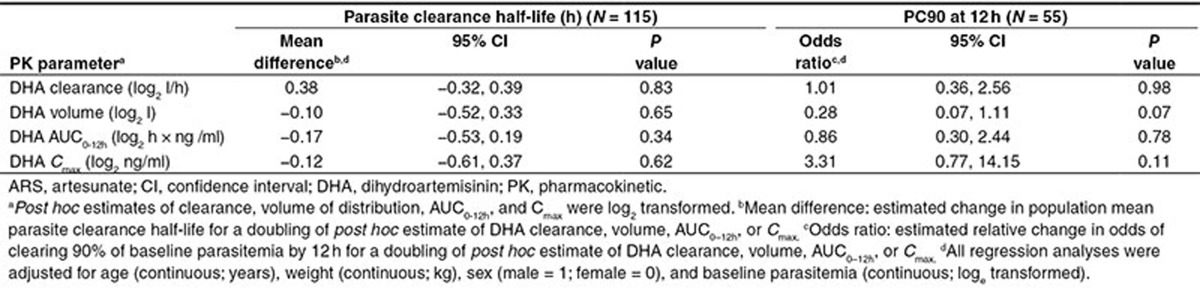
Estimates of association between the pharmacodynamic outcomes, parasite clearance half-life, and PC90 at 12 h, with the *post hoc* estimates of DHA clearance, volume of distribution, area under the curve (AUC_0–12h_), and maximum concentration (*C*_max_) derived from the final model fitted to the PK data from patients who received 2.4 mg/kg of i.v. ARS at baseline

**Table 5:**
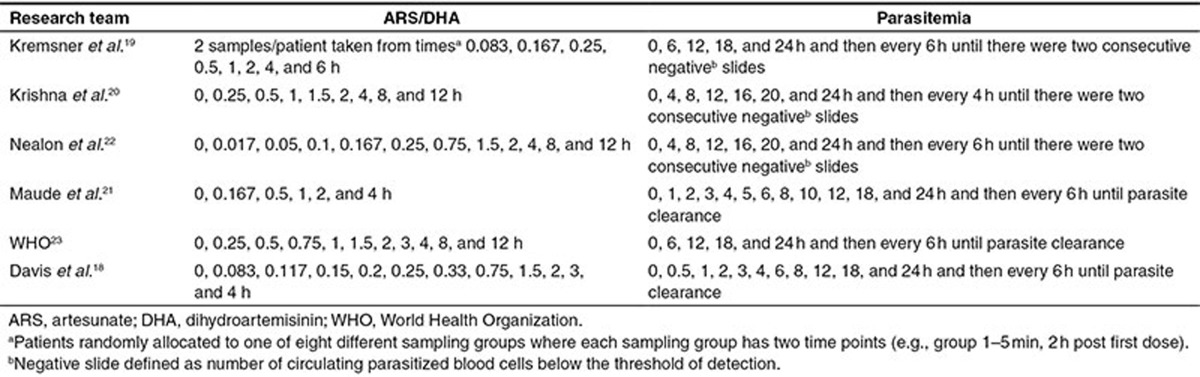
Sampling times for ARS/DHA concentrations and parasitemia measurements
