# Supplementary material for: Population Pharmacokinetics of Intravenous Artesunate: A Pooled Analysis of Individual Data From Patients With Severe Malaria
Source: CPT Pharmacometrics Syst Pharmacol. 2014 Nov 5;3(11):e145–. doi: 10.1038/psp.2014.43 (PMC4259998; doi:10.1038/psp.2014.43)
Supplement: Supplementary Information [file psp201443x1.pdf]

Supplementary Information

This document contains the supplementary information, supplementary figures and supplementary tables referred to in the main manuscript titled *'Population Pharmacokinetics of intravenous artesunate: a pooled analysis of individual data from patients with severe malaria'*.

Supplementary Information 1

Dihydroartemisinin (DHA) exposures (area under the curve, $AUC_{0\text{-}12h}$ h×ng/mL) after the standard 2.4 mg/kg dose were simulated at each body weight level (1000 simulations each at 1 kg intervals from 6 to 25 kg) using weight dependent uniform distributions of age (years; 6-10kg: 0.57,2.89; 11-15kg: 1.45,5.88; 16-20kg: 2.41,9.26; 21-25kg: 5.45,9.09) and haemoglobin (g/dL; 6-10kg: 2.4,12.5; 11-15kg: 4.33,13.3; 16-20kg: 4.33-11.8; 21-25kg: 8.6,11.3) and a weight independent uniform distribution for temperature (°C; 35-40.1) (Figure 2).

DHA exposures after the standard 2.4 mg/kg dose were simulated for patients older than 16 years at body weights between 33 kg and 75 kg (1000 simulations each at 3 kg intervals from 33 to 75 kg) using weight dependent uniform distributions of age (years; 33-44kg for 16,28; 45-50kg: 16,27; 51-60kg: 18,41; 61-75kg: 21,65) and haemoglobin (g/dL; 33-44kg: 2.9,13.8; 45-50kg: 6,13.1; 51-60kg: 5.1-13.2 ; 61-75kg: 6.5,15) and a weight independent uniform distribution for temperature (^{0}C; 36.6-40) (Figure 2; lower panel).

Supplementary Information 2

In brief the meta-analytic approach involved analysing the pharmacokinetic (PK) data from each of the six studies [1, 2, 3, 4, 5, 6], and each period of the cross-over trials, separately. Nonlinear mixed-effects (NLME) models were fitted to the individual datasets and an appropriate structural model (one or two distribution compartments) and error model (additive or

combined additive and proportional) was determined for each dataset. Before the PK data from the six studies were pooled each PK dataset, as well as PK datasets from each period of the cross-over trials, was analysed separately to determine whether the same structural model and error model is appropriate for all datasets.

Structural model selection. A NLME model with either a one-compartment or two-compartment IV bolus structural model and additive error on the natural log-scale were fitted to each dataset. Initially both NLME models were fitted by first order conditional estimation (FOCE) with interaction and assuming no below quantification limit (BQL) samples. In order to fit the NLME with a two-compartment structural model to each dataset, no covariance between the individual parameters was estimated and the variance of the following individual parameters was set to zero (i.e. assumed not to vary between patients) for the following studies:

- variance of the inter-compartmental clearance was fixed at zero for all studies;
- variance of the volume of distribution of the central compartment was fixed at zero for studies Kremsner *et al.* 2012 [2] and period two of Krishna *et al.* 2001 [3] ; and
- variance of the volume of distribution of the peripheral compartment was fixed at zero for period one of Nealon *et al.* 2002 [5].

Based on the visual predictive checks (VPCs), it was decided that the simpler one-compartment structural model captures the central tendency of the observations adequately and would be appropriate for all datasets.

The fit of the IV bolus one compartment model (which assumes instantaneous absorption) was then compared to a one compartment model with zero order absorption for each dataset. In order to fit the one compartment model with zero order absorption, absorption duration was assumed not to vary between patients and only the population average absorption parameter could be estimated. For all studies except Kremsner *et al.* 2012 [2], either the standard error of

the population pharmacokinetic (PK) parameter estimates could not be calculated if the population average absorption parameter was included in the model or the standard error of the population mean absorption duration parameter was very small (indicating lack of convergence of NONMEM's covariance step). The estimate of the absorption duration was also very sensitive to the initial value. The objective function value was similar for both models or tended to be greater for the zero absorption model and the population mean clearance and volume of distribution estimates were similar. Due to these fitting issues and the similarity of the population PK parameter estimates from the zero absorption model to the IV bolus model it was decided not to pursue to the zero absorption model.

Error model selection. Next NLME models with a one-compartment structural model and either an additive error on the natural log-scale or combined error model were fitted to each PK dataset. The additive error on the natural log-scale model was assumed appropriate for all datasets based on the VPCs.

Allometric scaling and BQL samples. Allometric scaling of the population mean PK parameters to be for a patient of median weight (15kg) further improved model fit according to the objective function value and VPCs. To examine if modelling below quantification limit (BQL) samples influences parameter estimation the NLME model with one-compartment structural model, exponential error model and body weight as an allometric function on the population mean clearance (CL) and volume of distribution (V) was fitted using the M3 method. The M3 method maximises the likelihood for the data above the limit of quantification (LOQ) and treats BQL concentrations as censored. The number of BQL concentrations for each study is given in Supplementary Table 4, along with the assay method and LOQ for the assay. The population mean PK parameter estimates derived by FOCE with interaction without any assumed BQL samples were similar to those derived by the M3 method for BQL samples and consequently, the VPCs were comparable to those produced

using the parameter estimates derived from the simplified estimation procedure, which assumes no BQL samples.

Meta-analytic approach. The meta-analytic approach involved comparing the population mean CL and V estimates for each study and period of the cross-over trials using a forest plot (Supplementary Figure 2). The population mean CL and V estimates plotted in Supplementary Figure 2 were derived by fitting a NLME model with one-compartment structural model, exponential error and body weight as an allometric function on the population mean CL and V parameters using the M3 method for BQL concentrations.

Supplementary Information 3

A 3-level Bayesian hierarchical model was fitted to the pooled pharmacokinetic (PK) data, which includes an extra level to account for between-study variability, in addition to the nonlinear mixed-effects (NLME) model which characterizes the within-patient processes of distribution and elimination, and the degree to which these processes vary between patients. The fit of this model was compared to the fit of a 2-level model including study as a fixed effect on both population mean clearance (CL) and volume of distribution (V) parameters, and a 2-level model that did not account for between-study variability. For each model, the residual variability was allowed to vary with study. The mathematical forms of the three hierarchical models fitted to the pooled PK data are given in Supplementary Table 5.

Bayesian estimation: In Bayesian statistics parameter estimates and interval estimates for each NLME model parameter are derived from the posterior distribution, which is the probability distribution of the NLME model parameters (population PK, individual PK and between-subject variability (BSV) parameters) given the data. Markov chain Monte Carlo (MCMC) methods were used to sample population PK, individual PK and BSV parameter values from the posterior distribution. MCMC algorithms were implemented in the open source software

packages WinBUGS 1.4.3 and R 3.0.1. Prior distributions for each parameter are described in Supplementary Table 5. For each NLME model parameter, WinBUGS was used to sample 100,000 parameter values from the posterior distribution. A burn-in of the first 50,000 samples was discarded and every 50th sample from the remaining 50,000 was retained, resulting in 1000 samples per parameter for calculation of parameter estimates and interval estimates. Parameter estimates are the median of the 1000 samples for each parameter (*posterior median*) and interval estimates are the 2.5^{th} and 97.5^{th} quantiles of the 1000 samples for each parameter, which is known as a 95% *credible interval* in Bayesian statistics (i.e. an interval in which the probability that the unknown parameter lies within is 0.95). Trace plots were examined to informally assess whether the 1000 samples per NLME model parameter were sampled from the posterior distribution (i.e. whether the MCMC algorithms had converged to the posterior distribution of the NLME model parameters).

Posterior predictive check: The fit of the 3-level model was examined using a posterior predictive check (PPC). The difference between a PPC and a visual predictive check (VPC) is the replicated datasets are simulated from the posterior predictive distribution (the distribution of replicated datasets given the observed dataset) for a PPC, while the replicated datasets for a VPC are generated by simulating under the model at the ML estimates for the population PK parameters. The visual display of the PPC for the 3-level hierarchical model is presented in Supplementary Figure 3, and is used analogously to a VPC to diagnose lack of model fit (e.g. if the model fits the data well then the observed percentiles should be contained in the 95% credible intervals for the corresponding predicted percentile). To generate replicated datasets from the posterior predictive distribution for the 3-level model, the 1000 parameter values sampled from the posterior distribution for the population mean clearance (CL) and volume of distribution (V) (averaged across studies) and between-study variability (Ω) (along with each patient's covariate information and sampling times) were used to simulate population PK parameters for each study (CL_k and V_k), individual PK parameters (CL_{ki} and V_{ki}) and

concentrations from level 1 of the 3-level hierarchical model (see Supplementary Table 5 for model definitions and corresponding table footnotes for parameter definitions).

Supplementary Information 4

Inclusion of covariates: The mathematical form of the individual clearance (CL_i) and volume (V_i) functions are given in equation (1) and (2), respectively.

$$CL_i = CL_{pop} \times (weight_i/weight_{pop})^{0.75} \times \textit{maturation function} \times \exp(\eta_{CL,i}) \quad (1)$$

$$V_i = V_{pop} \times (weight_i/weight_{pop}) \times \exp(\eta_{V,i}) \quad (2)$$

In equations (1) and (2), CL_{pop} and V_{pop} represent the population mean clearance and volume of distribution of DHA for the population investigated in a particular subgroup analysis; $weight_{pop}$ is set to 15kg for Subgroup analyses 1-3 (the median weight of the adults and children in Subgroup 1) and to 50kg for Subgroup 4 (the median weight of adults in this subgroup); *maturation function* is an age related enzyme-maturation effect (see [7, 8] for the full mathematical expression and a detailed explanation); $\eta_{CL,i}$ and $\eta_{V,i}$ are the inter-individual variability (IIV) for CL and V for the ith patient and are assumed to be normally distributed with mean 0 and variance ω^2. Correlation between the IIV for CL and V was also evaluated and represented by $\rho_{CL,V}$.

Stepwise covariate selection: For each subgroup analysis, potential covariates were investigated by adding covariates to their respective base models using a stepwise forward addition (p-value of 0.05) and backward elimination (p-value of 0.01) approach. Continuous covariates were included in equations (1) and (2) as linear functions, whose form is given in equations (3). The assumption of linearity was examined using plots of the post-hoc estimates versus covariate values and also with the likelihood ratio test.

$$\theta_i = \theta_{pop,i} \times (1 + \beta_{\theta,\,COV} \times (COV_i - \text{medianCOV})) \times \exp(\eta_{\theta,i}). \quad (3)$$

In equations (3), θ_i represents either the individual CL or V parameter for patient i; $\theta_{pop,i}$ represents either the population mean clearance or volume of distribution after scaling by body weight and age (see equations (1) and (2)); if COV_i is a continuous covariate centred at its median (medianCOV), then $\beta_{\theta,\,COV}$ is the fractional change in θ with each unit change from the median covariate value; if COV_i is cateogrical, then $\beta_{\theta,\,COV}$ is the fractional change in θ from the reference category to the non-reference categories; $\eta_{\theta,i}$ is the IIV for θ. Covariates found to be statistically significantly associated with the population mean PK parameters in Subgroup 1, were added to the base model for the stepwise covariate selection procedure in the subsequent subgroup analyses. The fit of the final models was examined using VPCs.

Supplementary Tables

Supplementary Table S1: Parameter estimates (relative standard error) for final models resulting from each subgroup analysis.

	**Subgroup 1**	**Subgroup 2**	**Subgroup 3**	**Subgroup 4**
Number of samples	1014	924	482	342
Number of patients	266	255	191	50
	Population estimate (%RSE)[1]	Population estimate (%RSE)	Population estimate (%RSE)	Population estimate (%RSE)
Fixed effects				
Population mean CL (L/h)[2]	12.99 (4.80)	12.84 (4.97)	11.69 (4.77)	40.18 (9.27)
Population mean V(L)[2]	11.56 (4.91)	11.56 (4.91)	11.26 (5.93)	44.31 (10.91)
$\beta_{V,MALE}$	-0.14 (37.39)	-0.14 (36.69)	-0.13 (46.24)	-0.29 (43.73)
Random effect standard deviation (sd) and correlation (cor)[3]				
sd of η_{CL}	0.35 (17.85)	0.35 (19.77)	0.28 (22.03)	0.41 (22.07)
sd of η_V	0.16 (28.36)	0.16 (32.94)	0.12 (31.59)	0.23 (28.63)
cor between η_{CL} and η_V	0.19 (31.15)	0.19 (46.74)	0.16 (21.15)	0.26 (24.51)
Residual standard deviation				
σ_1	0.34 (34.33)	0.34 (37.16)	0.37 (22.46)	

σ_2	0.36 (18.39)	0.37 (19.66)	0.36 (18.81)	
σ_3	0.43 (15.42)	0.43 (17.88)	0.46 (22.75)	
σ_4	0.36 (16.10)	0.38 (17.35)		0.39 (17.77)
σ_5	0.30 (14.92)	0.30 (15.09)		0.31 (17.45)
σ_6	0.27 (14.84)			

CL – DHA clearance (L/h); V – DHA volume of distribution (L); $\beta_{CL,MALE}$ – fractional change in population mean V for males compared to females; σ_i – residual standard deviation for study i =1 (Kremsner), 2 (Krishna), 3 (Nealon), 4 (Maude), 5 (WHO), 6 (Davis)
[1]Maximum likelihood estimate and %RSE calculated from asymptotic standard error; [2]For Subgroups 1-3 the population mean clearance (CL) and volume (V) values are for a female patient weighing 15kg. For Subgroup 4 the population mean clearance (CL) and volume (V) values are for a female patient weighing 50kg. [3]%RSE are for variance and covariance estimates.

Supplementary Table S2: Clinical features defined by the WHO and corresponding definition used by each study to classify patients as suffering from severe malaria.

WHO Definition	Research Team					
	Kremsner et al. [1]	Krishna et al.[2]	Nealon et al. [1]	Maude et al. [1]	WHO[2,3]	Davis et al.[2]
**Clinical features**						
Coma	N/A	BCS ≤ 2, with coma persisting for at least 1/2 h after the last seizure	BCS ≤ 2	GCS < 11	Examined, but no definition given	GCS < 11
Prostration	Examined – no definition given	N/A	N/A	N/A	Examined – no definition given	N/A
Failure to feed/Vomiting	Severe vomiting	N/A	N/A	N/A	N/A	N/A
Convulsions	N/A	N/A	Three or more observed convulsions	N/A	More than two in 24 hrs	N/A
Repiratory distress	Examined – no definition given	N/A	N/A	N/A	N/A	N/A
Shock	N/A	N/A	N/A	Systolic blood pressure < 80 mmHg with cool extremities	Systolic blood pressure < 70 mmHg with cool extremities	N/A
Jaundice	Visible or serum bilirubin ≥ 3mg/dL	N/A	N/A	Bilirubin > 3.0 mg/dL with parasitaemia > 100,000/µL	Serum bilirubin ≥ 3mg/dL	Serum bilirubin level > 50 µmol/L; Serum aspartate transaminase > twice upper

						limit of the reference range
Haemoglobinuria	Dipstick positive dark urine	N/A	N/A	N/A	Examined – no definition given	N/A
Abnormal spontaneous bleeding	N/A	N/A	N/A	N/A	Examined – no definition given	N/A
Pulmonary oedema	N/A	N/A	N/A	N/A	Examined – no definition given	N/A
**Laboratory findings**						
Hypoglycemia	Glucose < 2.2 mmol/L	Capillary or venous glucose concentration ≤ 2.2 mmol/L	Glucose concentration in whole blood or capillary blood of ≤ 2.2 mmol/L	Blood glucose < 40 mg/dL	Glucose concentration in whole blood ≤ 2.2 mmol/L	N/A
Metabolic acidosis	N/A	N/A	N/A	Plasma venous bicarbonate < 15 mmol/L	Plasma venous bicarbonate < 15 mmol/L	N/A
Anaemia	No threshold given	Packed cell volume < 15%	Haemoglobin < 5 g/dl and/or haematocrit < 15%	Haematocrit < 20% with parasitaemia > 100 000/µL	Haemoglobin < 5 g/dl and/or haematocrit < 15%	Venous haematocrit < 15%
Haemoglobinuria	N/A	N/A	N/A	N/A	Examined – no definition given	N/A
Parasitaemia	Parasitaemia ≥ 10%	N/A	N/A	Parasitaemia > 10 %	Examined – no definition given	Parasitaemia > 250 000/µL
Hyperlactaemia	Lactate	Capillary or	Lactate	Venous plasma	N/A	N/A

	> 5 mmol/L	venous lactate concentration ≥ 5 mmol/L	concentration in whole blood or capillary blood of ≥ 5 mmol/L	lactate > 4 mmol/L		
Renal impairment	N/A	N/A	N/A	Serum creatinine > 3 mg/dL	Serum creatinine > 3 mg/dL	Serum creatinine level > 250 μmol/L after rehydration

[1]Inclusion criteria for study; [2]Exclusion criteria for study; [3]Internal WHO reports states that severe malaria defined according to 1990 WHO criteria (see Severe and complicated malaria. World Health Organization, Division of Control of Tropical Diseases. *Trans R Soc Trop Med Hyg* 1990, **84 Suppl 2:** 1-65).

Supplementary Table S3: Lists the assay method, limit of quantification (LOQ) and limit of detection (LOD) of the assay, and laboratory where the assay was performed.

Research team	Assay method*	LOQ/LOD (ng/mL)	Laboratory
Kremsner et al.	LC – MS	LOQ – 2.84<br>LOD – 0.28	AFFRIMS, Bangkok, Thailand
Krishna et al.	HPLC – ECD	LOQ –50<br>LOD – 8	Centre for Drug Research, University Sains Malaysia, Malaysia
Nealon et al.	HPLC – ECD	LOQ –50<br>LOD – 8	Parasitology Dept, Marseille Armees, France
Maude et al.	LC – MS / MS	LOQ – 2<br>LOD – 0.6	Clinical Pharmacology Laboratory, MORU, Bangkok, Thailand
WHO	HPLC – ECD	LOQ –50<br>LOD –8	Centre for Drug Research, University Sains Malaysia, Malaysia
Davis et al.	HPLC – UVD	LOQ –42.6<br>LOD –20	Department of Pharmacology, University of Western Australia, Australia

*ECD – electrochemical detection; MS – mass spectrometry; LC – liquid chromatography; HPLC – high performance liquid chromatography; UVD - ultraviolet detection

Supplementary Table S4: Description of DHA sampling for each study.

	**Children**			**Adults**			
	**Kremsner**	**Krishna**	**Nealon**	**Maude**	**WHO**	**Davis**	**Total**
Assay method*	LC – MS	HPLC – ECD	HPLC –ECD	LC – MS/MS	HPLC –ECD	HPLC – UVD	n/a
Limit of Quantification (LoQ)	2.84	50	50	2	50	42.6	n/a
No. patients	179	29	19	18	48	7	317
No. samples (%Below LoQ)	526 (2.47%)	159 (6.92%)	150 (2.00%)	113 (0.88%)	409 (6.6%)	88 (0%)	1552 (3.61%)
Median No.samples / patient [Range]	2 [1-3]	5 [1-7]	7 [4-9]	5 [3-7]	8 [3-11]	11 [10-13]	2 [1-13]

*ECD – electrochemical detection; MS – mass spectrometry; LC – liquid chromatography; HPLC – high performance liquid chromatography; UVD - ultraviolet detection

Supplementary Table S5: Mathematical form of hierarchical models fitted to the pooled PK data to investigate whether modeling between-study differences in the population mean PK parameters influenced the predictive properties of the model.

	Model[ǂ]		
**Level**	**No study (2-level)**	**Study Fixed (2-level)**	**Study Random (3-level)**
1. Within-patient	$\log y_{kij} \sim N(\log f(D_{ki}, t_{kij}; \theta_{ki}), \sigma_k^2)$		
2. Between-patient	$\theta_i \sim N_2(\theta, \Sigma)$	$\theta_{ki} \sim N_2(\theta_k, \Sigma)$	
3. Between-study	n/a	n/a	$\theta_k \sim t_{2,\nu}(\theta, \Omega)$
Prior[¥]	$\sigma_k^{-2} \sim \Gamma(a, b)$<br>$\theta \sim N_2(m, P^{-1}\times I)$<br>$\Sigma^{-1} \sim W_2(S_1, \nu_1)$	$\sigma_k^{-2} \sim \Gamma(a, b)$<br>$\theta_k \sim N_2(m, P^{-1}\times I)$<br>$\Sigma^{-1} \sim W_2(S_1, \nu_1)$	$\sigma_k^{-2} \sim \Gamma(a, b)$<br>$\theta \sim N_2(m, P^{-1}\times I)$<br>$\Sigma^{-1} \sim W_2(S_1, \nu_1)$<br>$\Omega^{-1} \sim W_2(S_2, \nu_2)$

[ǂ]k^{th} – study; i^{th} – individual; j^{th} – concentration; *f* – one-compartment model; D_{ki} – administered dose of DHA; t_{kij} – sampling time (hours); σ_k^2 –residual variance or within-patient variability; $\theta_{ki} = [\log CL_{ki}, \log V_{ki}]'$; $\theta_k = [\log CL_k, \log V_k]'$; $\theta = [\log CL, \log V]'$; CL – clearance; V – volume of distribution; Σ – between patient variability; Ω – between study variability; N – normal distribution; N_2 – bivariate normal distribution; $t_{2,\nu}$ – bivariate student t-distribution with ν degrees of freedom; Γ –gamma distribution; W_2 – bivariate Wishart distribution; I – identity matrix

[¥]$a = b = 0.001$; $m=0$; $P^{-1}=10^{-4}$; $S_1= S_2=\sinh^{-1}(0.1) \times I$; $\nu_1= \nu_2=2$

Supplementary Table S6: Method of parasitaemia measurement for each study included in the pooled analysis.

Study*	Report of parasitaemia measurement in paper
Kremsner *et al.* 2012 [19]	Smears were prepared by the Lambarene method and read by 2 microscopists independently (Planche *et al.* 2001**)
Krishna *et al.* 2001 [20]	Parasitaemia was confirmed and quantitated using Field's stain (by counting the number of parasites per 1000 RBCs on a thin film or per 200 WBCs on a thick film) by an experienced microscopist.
Nealon *et al.* 2002 [22]	Thick and thin blood films were stained with Giemsa of Field's stain, respectively; and counts were obtained as described before (Krishna *et al.* 2001 [20]; Planche *et al.* 2001**).
Maude *et al.* 2009 [21]	Thick and thin films with parasites per 100 RBCs on thin films and per 200 and 500 WBCs on thin films. Field stain was used.
WHO (subset published in Simpson *et al.* 2006 [23])	Thick and thin blood films stained with reverse Field's stain and expressed per 1000 RBCs or 200 WBCs. Slides read by experienced microscopist.
Davis *et al.* 2001[18]	Thick and thin blood films were prepared.

RBC – red blood cell; WBC – white blood cell

*Superscripts are references provided in main text.

** Planche T, Krishna S, Kombila M, et al. Comparison of methods for the rapid laboratory assessment of children with malaria. Am J Trop Med Hyg 2001; 65:599–602.

Supplementary Figures

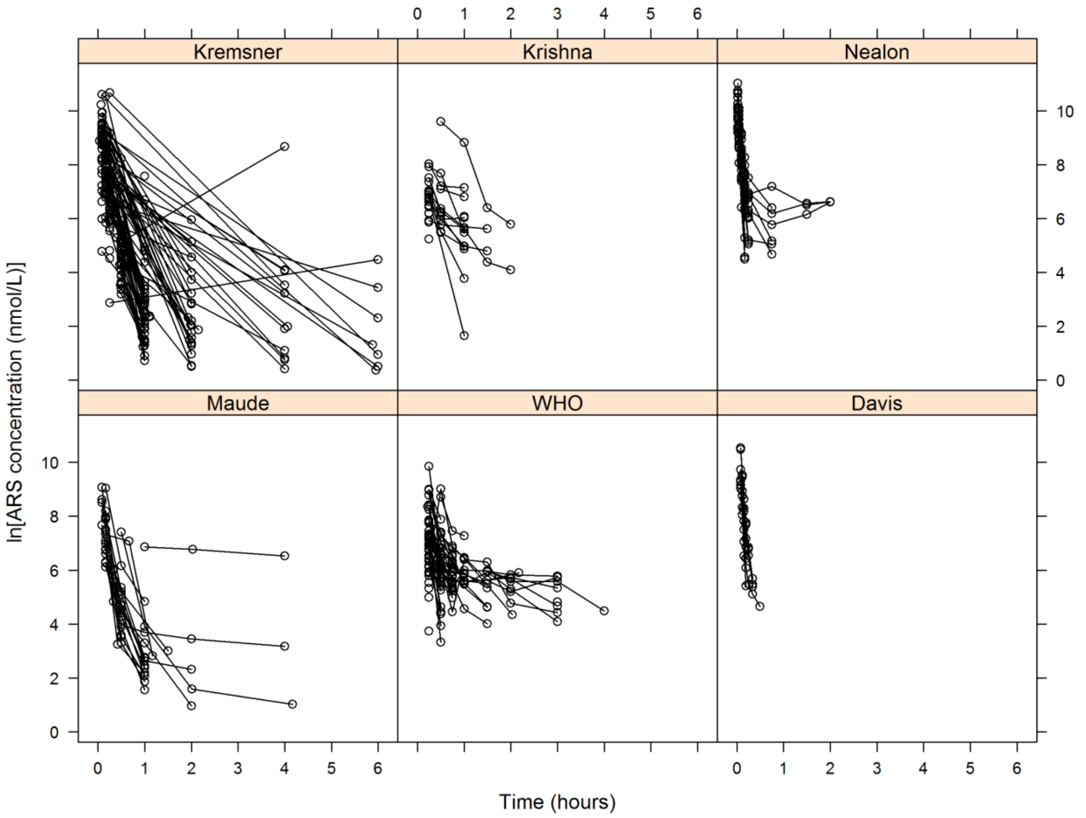

Supplementary Figure S1: ARS concentration (natural log-transformed; nmol/L) data versus sampling time (hrs) are plotted for each of the six studies. Five of the studies are referred to by the first author of the paper the data were published in (Kremsner *et al.* 2012 [2], Krishna *et al.* 2001 [3], Nealon *et al.* 2002 [5] Maude *et al.* 2009 [4] and Davis *et al.* 2001 [1]) and the data from the WHO[6] are referred to as WHO.

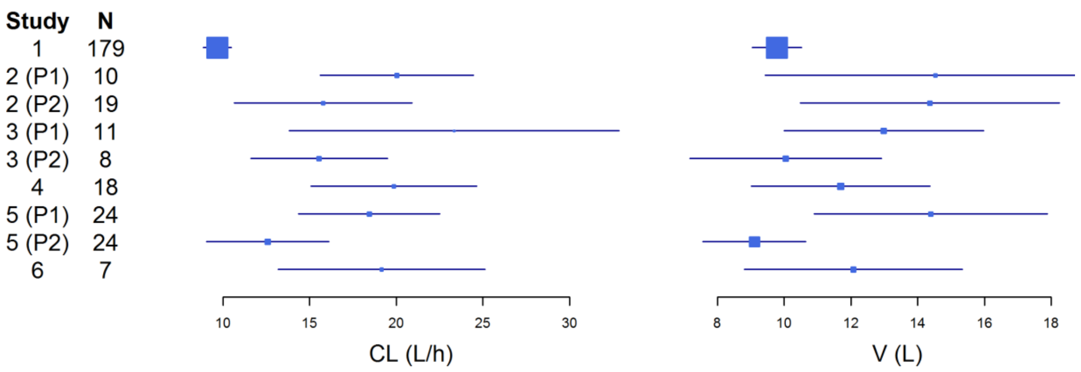

Supplementary Figure S2: Forest plots of population mean clearance (CL) estimates and population mean volume of distribution (V) estimates for each dataset, derived by using the M3 method to fit a nonlinear mixed-effects (NLME) model with one-compartment structural model, exponential error model and body weight as an allometric function on population mean PK parameters. Population mean CL and V parameters were allometrically scaled by body weight to be for a patient weighing 15kg as described in section Supplementary Information 4. The blue boxes are parameter estimates and the size is proportional to the inverse of the standard error of the estimate squared. Horizontal blue lines represent 95% confidence intervals for the population parameter.

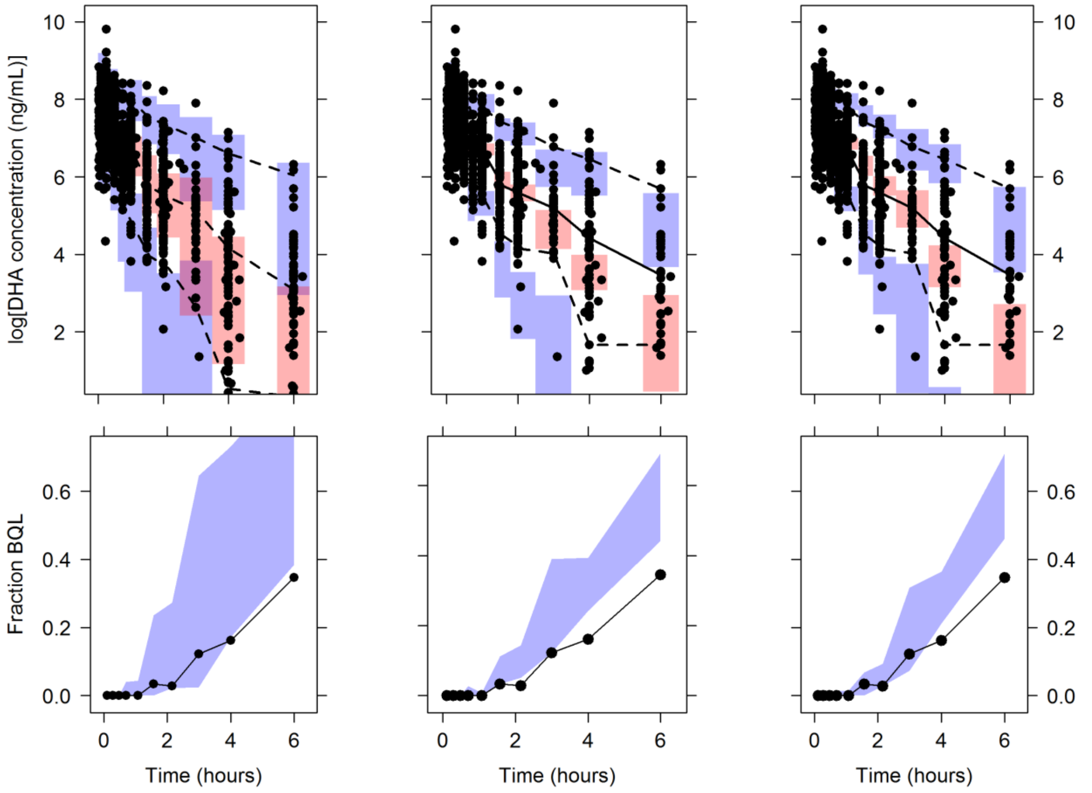

Supplementary Figure S3: Posterior predictive check (PPC) of 3-level hierarchical model (far left panel). Visual predictive checks (VPC) of model including a fixed study effect (middle panel) and model with no study effect (far right panel). The top panel of VPCs are for the continuous concentrations-time data and the bottom panel of VPCs are for the fraction of concentration below the limit of concentration.

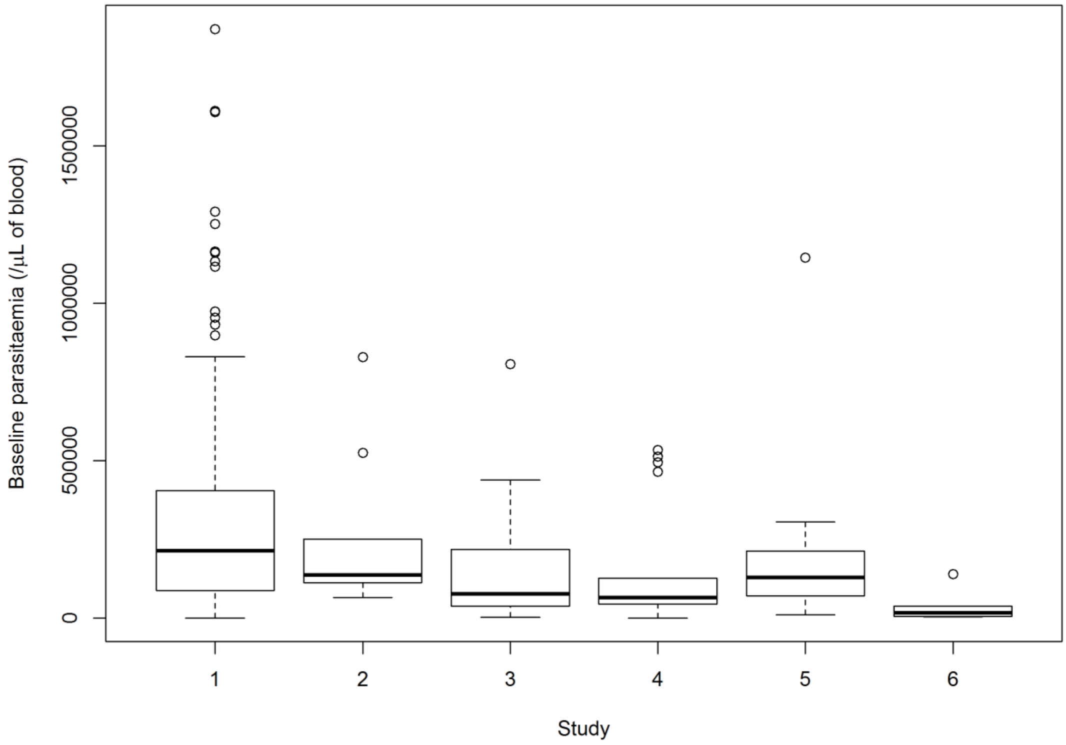

Supplementary Figure S4: Boxplots of baseline parasitaemia (/µL of blood) from 142 patients included in the analysis of the parasitological outcomes stratified by study. Study 1 = Kremsner *et al.* 2012 [19] (N=80); 2 = Krishna *et al.* 2001 [20] (N=10); 3 = Nealon *et al.* 2002 [22] (N=9); 4 = Maude *et al.* 2009 [21] (N=17); 5 =WHO (subset published in Simpson *et al.* 2006 [23]; N=21); 6 = Davis *et al.* 2001[18] (N=5). Note superscripts are references provided in the main text.

References

1. Davis TM, Phuong HL, Ilett KF, Hung NC, Batty KT, Phuong VD*, et al.* Pharmacokinetics and pharmacodynamics of intravenous artesunate in severe falciparum malaria. *Antimicrobial agents and chemotherapy* 2001, **45**(1)**:** 181-186.

2. Kremsner PG, Taylor T, Issifou S, Kombila M, Chimalizeni Y, Kawaza K*, et al.* A simplified intravenous artesunate regimen for severe malaria. *The Journal of infectious diseases* 2012, **205**(2)**:** 312-319.

3. Krishna S, Planche T, Agbenyega T, Woodrow C, Agranoff D, Bedu-Addo G*, et al.* Bioavailability and preliminary clinical efficacy of intrarectal artesunate in Ghanaian

children with moderate malaria. *Antimicrobial agents and chemotherapy* 2001, **45**(2)**:** 509-516.

4. Maude RJ, Plewes K, Faiz MA, Hanson J, Charunwatthana P, Lee SJ*, et al.* Does artesunate prolong the electrocardiograph QT interval in patients with severe malaria? *The American journal of tropical medicine and hygiene* 2009, **80**(1)**:** 126-132.

5. Nealon C, Dzeing A, Muller-Romer U, Planche T, Sinou V, Kombila M*, et al.* Intramuscular bioavailability and clinical efficacy of artesunate in gabonese children with severe malaria. *Antimicrobial agents and chemotherapy* 2002, **46**(12)**:** 3933-3939.

6. Simpson JA, Agbenyega T, Barnes KI, Di Perri G, Folb P, Gomes M*, et al.* Population pharmacokinetics of artesunate and dihydroartemisinin following intra-rectal dosing of artesunate in malaria patients. *PLoS Med* 2006, **3**(11)**:** e444.

7. Hayton WL. Maturation and growth of renal function: dosing renally cleared drugs in children. *AAPS PharmSci* 2000, **2**(1)**:** 22-28.

8. Jamsen KM, Duffull SB, Tarning J, Lindegardh N, White NJ, Simpson Ja. Optimal designs for population pharmacokinetic studies of oral artesunate in patients with uncomplicated falciparum malaria. *Malaria journal* 2011, **10**(1)**:** 181-181.

NONMEM code: IV-ARS pooled pharmacokinetic analysis

1. NONMEM model code used to produce results in Table 3, column "All severe malaria patients"

```
$PROBLEM    IV-ARS pooled analysis
            (266 patients; include patients in period 2 of cross-over trials)
$INPUT
ID          ; Patient identifier
EVID        ; 1=dose event; 0=observation
AMT         ; IV-ARS dose (micrograms)
TIME        ; PK sampling time (hours)
DV          ; DHA blood plasma concentration (ng/mL)
STDY        ; Study identifier
AGE         ; age (years)
WGT         ; weight (kg)
SEX         ; 1=male; 0=female
TEMP        ; Body temperature (degrees Celsius)
IPL         ; Initial parasite load (/microliter of blood)
LOGIPL      ; Natural logarithm of IPL
HB          ; Haemoglobin (g/dL)
ADULT       ; 1=adult; 0=child
CENS        ; 1=below quantification limit (BQL); 0=above lower limit of
            ; quantification (LLOQ)
LLOQ        ; Lower limit of quantification for each study

$DATA       pooled_NM_logDV_omitNewton.txt IGNORE=@

$ABB COMRES=5
```

```
$SUBROUTINE ADVAN1

$PK

;;; VSEX-DEFINITION START
 IF(SEX.EQ.0) VSEX = 1
 IF(SEX.EQ.1) VSEX = 1 + THETA(9)
;;; VSEX-DEFINITION END

;;; V-RELATION START
 VCOV = VSEX
;;; V-RELATION END

 AGEmth = AGE*12    ; convert age (years) to months
 WGTmed = 15        ; center weight

;; Specify CL and V models
; CL & V allometrically scaled by weight
; CL includes an additional age maturation model

 ; Population PK parameters
 TVCL = THETA(1)
 TVV  = THETA(2)
 TVV  = VCOV*TVV

 ; Individual PK parameters
 CL = ((TVCL/3.1)*((WGT/WGTmed)**0.75)*EXP(-0.082*AGEmth) +
     TVCL*((WGT/WGTmed)**0.75)*(1-EXP(-0.082*AGEmth)))*EXP(ETA(1))

 V  = TVV*(WGT/WGTmed)*EXP(ETA(2))

 lnCL = log(CL)
 lnV  = log(V)

 K = CL/V
 S1 = V

$INFN

IF (ICALL.EQ.3) THEN
 OPEN(50,FILE='cwtab5.est')
 WRITE(50,*) 'ETAS'
 DO WHILE(DATA)
  IF (NEWIND.LE.1) WRITE (50,*) ETA
 ENDDO
 WRITE(50,*) 'THETAS'
 WRITE(50,*) THETA
 WRITE(50,*) 'OMEGAS'
 WRITE(50,*) OMEGA(BLOCK)
 WRITE(50,*) 'SIGMAS'
 WRITE(50,*) SIGMA(BLOCK)
ENDIF

$ERROR

;; Create new binary variables ST13 to ST9
 ; Allows residual variance to vary by study
 ST13 = 0
 ST4  = 0
 ST5  = 0
 ST6  = 0
 ST8  = 0
 ST9  = 0

 IF(STDY.EQ.1) ST13 = 1
 IF(STDY.EQ.2) ST13 = 1
 IF(STDY.EQ.3) ST13 = 1
```

```
 IF(STDY.EQ.4) ST4  = 1
 IF(STDY.EQ.5) ST5  = 1
 IF(STDY.EQ.6) ST6  = 1
 IF(STDY.EQ.8) ST8  = 1
 IF(STDY.EQ.9) ST9  = 1

; Predict IV-ARS concentrations
 IPRED = -5
 IF(F.GT.0) IPRED = LOG(F)

; Residual error model
 W     = (THETA(3)*ST13) +(THETA(4)*ST4)+ (THETA(5)*ST5) + (THETA(6)*ST6)
         + (THETA(7)*ST8) + (THETA(8)*ST9)

; Calculate residuals
 IRES  = DV - IPRED
 IF(W.EQ.0) W = 1
 IWRES = IRES/W

; Implement M3
 IF (DV>=LLOQ) THEN
  F_FLAG = 0
  Y = IPRED + W*EPS(1)
 ELSE
  F_FLAG=1
  Y = PHI((LLOQ-IPRED)/W)
 ENDIF

 IF (DV<LLOQ) THEN
  BQL = 0
 ELSE
  BQL = 1
 ENDIF

"LAST
"  COM(1)=G(1,1)
"  COM(2)=G(2,1)
"  COM(3)=HH(1,1)

;; Initial values
$THETA 13.0298      ; CL
       9.95126      ; V
       (0,0.335103) ; SIG1
       (0,0.359492) ; SIG2
       (0,0.585451) ; SIG3
       (0,0.48754)  ; SIG4
       (0,0.294651) ; SIG6
       (0,0.273582) ; SIG7
$THETA (-1000000,0.198519,1000000) ; VSEX1

$OMEGA  BLOCK(2)
 0.348113  ; var(CL)
 0.169903  ; cov(CL,V)
 0.21785   ; var(V)

$SIGMA  1  FIX  ; a

$ESTIMATION NOABORT MAXEVAL=5000 METHOD=COND INTER LAPLACIAN PRINT=5
$COVARIANCE PRINT=E

$TABLE       ID COM(1)=G11 COM(2)=G21 COM(3)=H11 IPRED MDV
             NOPRINT ONEHEADER FILE=cwtab5.deriv

$TABLE       ID TIME IPRED IWRES
             NOPRINT ONEHEADER FILE=sdtab5

$TABLE       ID CL V lnCL lnV ETA1 ETA2
             NOPRINT ONEHEADER FILE=patab5
```

```
$TABLE       ID STDY SEX CENS BQL ADULT
             NOPRINT ONEHEADER FILE=catab5

$TABLE       ID AGE WGT SEX TEMP IPL LOGIPL HB LLOQ
             NOPRINT ONEHEADER FILE=cotab5

$TABLE       ID
             NOPRINT ONEHEADER FILE=cwtab5.est
```

2. **NONMEM model code used to produce results in Table 3, column "Only received IV-ARS at baseline"**

```
$PROBLEM    IV-ARS pooled analysis
            (223 patients; exclude patients in period 2 of cross-over trials)

$ABBREVIATED COMRES=5

$INPUT       ID EVID AMT TIME DV STDY STDYPER AGE WGT SEX TEMP IPL LOGIPL HB
             ADULT CENS LLOQ

ID           ; Patient identifier
EVID         ; 1=dose event; 0=observation
AMT          ; IV-ARS dose (micrograms)
TIME         ; PK sampling time (hours)
DV           ; DHA blood plasma concentration (ng/mL)
STDY         ; Study identifier
STDYPER      ; Study identifier (distinguish between periods of cross-over
             ; trials)
AGE          ; age (years)
WGT          ; weight (kg)
SEX          ; 1=male; 0=female
TEMP         ; Body temperature (degrees Celsius)
IPL          ; Initial parasite load (/microliter of blood)
LOGIPL       ; Natural logarithm of IPL
HB           ; Haemoglobin (g/dL)
ADULT        ; 1=adult; 0=child
CENS         ; 1=below quantification limit (BQL); 0=above lower limit of
             ; quantification (LLOQ)
LLOQ         ; Lower limit of quantification for each study


$DATA       pooled_NM_logDV_omitNewton_rmP2.txt IGNORE=@

$SUBROUTINE ADVAN1

$PK

;;; VSEX-DEFINITION START
IF(SEX.EQ.0) VSEX = 1
IF(SEX.EQ.1) VSEX = 1 + THETA(11)
;;; VSEX-DEFINITION END

;;; V-RELATION START
VCOV=VSEX
;;; V-RELATION END


;;; CLTEMP-DEFINITION START
CLTEMP = ( 1 + THETA(10)*(TEMP - 38.20))
;;; CLTEMP-DEFINITION END


;;; CLHB-DEFINITION START
CLHB = ( 1 + THETA(9)*(HB - 8.80))
;;; CLHB-DEFINITION END

;;; CL-RELATION START
CLCOV=CLHB*CLTEMP
```

```
;;; CL-RELATION END


 AGEmth = AGE*12    ; convert age (years) to months
 WGTmed = 15        ; center weight

;; Specify CL and V models
 ; CL & V allometrically scaled by weight
 ; CL includes an additional age maturation model

 ; Population PK parameters
 TVCL = THETA(1)
 TVCL = CLCOV*TVCL
 TVV = THETA(2)
 TVV = VCOV*TVV

 ; Individual PK parameters
 CL = ((TVCL/3.1)*((WGT/WGTmed)**0.75)*EXP(-0.082*AGEmth)
      + TVCL*((WGT/WGTmed)**0.75)*(1-EXP(-0.082*AGEmth)))*EXP(ETA(1))

 V = TVV*(WGT/WGTmed)*EXP(ETA(2))

 lnCL = log(CL)
 lnV = log(V)

 K = CL/V
 S1 = V

$INFN

IF (ICALL.EQ.3) THEN
 OPEN(50,FILE='cwtab4.est')
 WRITE(50,*) 'ETAS'
 DO WHILE(DATA)
  IF (NEWIND.LE.1) WRITE (50,*) ETA
 ENDDO
 WRITE(50,*) 'THETAS'
 WRITE(50,*) THETA
 WRITE(50,*) 'OMEGAS'
 WRITE(50,*) OMEGA(BLOCK)
 WRITE(50,*) 'SIGMAS'
 WRITE(50,*) SIGMA(BLOCK)
ENDIF

$ERROR

;; Create new binary variables ST13 to ST9
 ST13 = 0
 ST4  = 0
 ST5  = 0
 ST6  = 0
 ST8  = 0
 ST9  = 0

 IF(STDY.EQ.1) ST13 = 1
 IF(STDY.EQ.2) ST13 = 1
 IF(STDY.EQ.3) ST13 = 1
 IF(STDY.EQ.4) ST4  = 1
 IF(STDY.EQ.5) ST5  = 1
 IF(STDY.EQ.6) ST6  = 1
 IF(STDY.EQ.8) ST8  = 1
 IF(STDY.EQ.9) ST9  = 1

 ; Predict IV-ARS concentrations
 IPRED = -5
 IF(F.GT.0) IPRED = LOG(F)

 ; Residual error model
```

```
 W = (THETA(3)*ST13) + (THETA(4)*ST4) + (THETA(5)*ST5) + (THETA(6)*ST6)
         + (THETA(7)*ST8) + (THETA(8)*ST9)

; Calculate residuals
IRES  = DV - IPRED
IF(W.EQ.0) W = 1
IWRES = IRES/W

; Implement M3
IF (DV>=LLOQ) THEN
 F_FLAG=0
 Y = IPRED + W*EPS(1)
ELSE
 F_FLAG=1
 Y=PHI((LLOQ-IPRED)/W)
ENDIF

IF (DV<LLOQ) THEN
 BQL = 0
ELSE
 BQL = 1
ENDIF

; Initial values
$THETA 12.8902      ; CL
       11.66        ; V
       (0,0.35186)  ; SIG1
       (0,0.35226)  ; SIG2
       (0,0.366449) ; SIG3
       (0,0.43839)  ; SIG4
       (0,0.33377)  ; SIG6
       (0,0.273717) ; SIG7

$THETA  (-0.161,0.1,0.156)       ; CLHB1
$THETA  (-0.370,0.1,0.25)        ; CLTEMP1
$THETA  (-1000000,0.1,1000000)   ; VSEX1

$OMEGA  BLOCK(2)
 0.307469  ; var(CL)
 0.139349  ; cov(CL,V)
 0.174615  ; var(V)

$SIGMA  1  FIX  ; a

$ESTIMATION NOABORT MAXEVAL=5000 METHOD=COND INTER LAPLACIAN PRINT=5
$COVARIANCE PRINT=E

$TABLE       ID COM(1)=G11 COM(2)=G21 COM(3)=H11 IPRED MDV
             NOPRINT ONEHEADER FILE=cwtab4.deriv

$TABLE       ID TIME IPRED IWRES
             NOPRINT ONEHEADER FILE=sdtab4

$TABLE       ID CL V lnCL lnV ETA1 ETA2
             NOPRINT ONEHEADER FILE=patab4

$TABLE       ID STDY SEX CENS BQL ADULT
             NOPRINT ONEHEADER FILE=catab4

$TABLE       ID AGE WGT SEX TEMP IPL LOGIPL HB LLOQ
             NOPRINT ONEHEADER FILE=cotab4

$TABLE       ID
             NOPRINT ONEHEADER FILE=cwtab4.est
```
